# Satisfaction after total knee arthroplasty: a prospective matched-pair analysis of patients with customised individually made and off-the-shelf implants

**DOI:** 10.1007/s00167-023-07643-1

**Published:** 2023-11-20

**Authors:** Nicole Vogel, Raphael Kaelin, Thomas Rychen, Séverin Wendelspiess, Magdalena Müller-Gerbl, Markus P. Arnold

**Affiliations:** 1 Practice MEIN KNIE, Hirslanden Klinik Birshof, Reinacherstrasse 42, 4142 Münchenstein, Switzerland; 2 Practice LEONARDO, Hirslanden Klinik Birshof, Münchenstein, Switzerland; 3 Faculty of Medicine, University of Basel, Basel, Switzerland; 4 Department of Biomedicine, University of Basel, Basel, Switzerland

**Keywords:** Total knee arthroplasty, Custom implant, Off-the-shelf implant, Patient-specific, Patient-reported outcome measure, Patient satisfaction, Matched-pair analysis

## Abstract

**Purpose:**

Customised individually made (CIM) total knee arthroplasty (TKA) was introduced to potentially improve patient satisfaction and other patient-reported outcome measures (PROMs). The purpose of this study was to compare PROMs, especially patient satisfaction, of patients with CIM and OTS TKA in a matched-pair analysis with a 2-year follow-up.

**Methods:**

This is a prospective cohort study with a propensity score matching of 85 CIM and 85 off-the-shelf (OTS) TKA. Follow-up was at 4 months, 1 year and 2 years. The primary outcome was patient satisfaction. Secondary outcomes were as follows: overall improvement, willingness to undergo the surgery again, Knee injury and Osteoarthritis Outcome Score (KOOS), Forgotten Joint Score (FJS-12), High-Activity Arthroplasty Score (HAAS), EQ-5D-3L, EQ-VAS, Knee Society Score (KSS) and surgeon satisfaction.

**Results:**

Patient satisfaction ranged from 86 to 90% and did not differ between CIM and OTS TKA. The EQ-VAS after 4 months and the HAAS after 1 year and 2 years were higher for CIM TKA. KOOS, FJS-12 and EQ-5D-3L were not different at follow-up. The changes in KOOS symptoms, pain and daily living were higher for OTS TKA. The KSS was higher for patients with CIM TKA. Surgeon satisfaction was high throughout both groups. Patients who were satisfied after 2 years did not differ preoperatively from those who were not satisfied. Postoperatively, all PROMs were better for satisfied patients. Patient satisfaction was not correlated with patient characteristics, implant or preoperative PROMs, and medium to strongly correlated with postoperative PROMs.

**Conclusion:**

Patient satisfaction was high with no differences between patients with CIM and OTS TKA. Both implant systems improved function, pain and health-related quality of life. Patients with CIM TKA showed superior results in demanding activities as measured by the HAAS.

**Level of evidence:**

II, prospective cohort study.

**Supplementary Information:**

The online version contains supplementary material available at 10.1007/s00167-023-07643-1.

## Introduction

Achieving a high percentage of satisfied patients after a total knee arthroplasty (TKA) is still challenging. Despite the success of TKA, about 20% of patients remain dissatisfied [1–3]. Several factors and predictors have been identified [2, 4–9], with persistent pain and limited function being the main reasons for patient dissatisfaction [10]. To better understand the patients’ perspective, the analysis of patient-reported outcome measures (PROMs), and patient satisfaction in particular, is inevitable. From a patient-centred perspective, a TKA is only successful if the patient is satisfied with the outcome.

Customised individually made (CIM) TKAs were introduced in 2011 [11]. CIM implants are manufactured based on a computed tomography scan of the affected leg. The underlying concept is to respect the anatomical variability and to restore the individual anatomy, thereby improving knee kinematics. Off-the-shelf (OTS) TKAs can cause implant overhang, malalignment and abnormal kinematics [12–15]. CIM TKAs were designed to overcome these limitations and to improve clinical outcome and patient satisfaction. The high variability in morphology supports the evolution towards CIM TKA to potentially achieve better bone–implant fit [16, 17].

Studies have shown encouraging results with CIM TKA regarding knee alignment [18, 19], improved function [20] and patient satisfaction [21, 22]. Recent systematic reviews found conflicting evidence with superior and inferior results for clinical and patient-reported outcomes with CIM TKA [23–25]. However, they highlighted the need for better methodological studies.

A prospective study of CIM TKA with a matched-pair control group focussing on PROMs is currently not published. The purpose of this study was to compare PROMs, especially patient satisfaction, of patients with CIM and OTS TKA in a matched-pair analysis with a 2-year follow-up. Our hypothesis was that patients with CIM TKA would have a higher rate of patient satisfaction than patients with OTS TKA.

## Materials and methods

### Study design, setting and recruitment

This is a single-side, observational, prospective cohort study with matched-pair analyses comparing patients with CIM and OTS TKA. The study was conducted in accordance with the World Medical Association Declaration of Helsinki [26] and approved by the local ethics committee (reference: 2016-01777).

Patients were recruited in our medical practise. Routinely, all of our TKA patients were asked to complete a set of PROMs. Details regarding recruitment and procedures are published elsewhere [27]. In brief, after signed consent, patients completed PROMs before the surgery, at 4 months, 1 year and 2 years. In the current study, we included consecutive patients with a primary cruciate-retaining CIM TKA (iTotal^®^ CR G2, Conformis Inc., Billerica, MA, US) or primary cruciate-retaining OTS TKA (Attune^®^ CR mobile-bearing, DePuy Synthes, Raynham, MA, US) who completed PROMs before the surgery and after 2 years. Patients were excluded if they had a major re-operation with potential impact on the TKA or revision.

### Implants and surgery technique

The CIM TKA implant is based on a preoperative computed tomography. The surgeon is provided with a customised implant and customised instruments. The concept and surgical technique are described elsewhere [28]. In brief, the distal femoral resection is performed using a patient-specific cutting block and the tibial resection is performed using a cutting jig for the patient-specific anatomical slope. Patient-specific spacers are used to balance the knee in extension and flexion. The planning algorithm aims for a hip–knee–ankle angle of 180° and a limited joint line obliquity due to uneven medial and lateral inlay heights.

The Attune implant used in the control group is the most commonly used OTS implant in Switzerland [29]. OTS TKA was performed with conventional instrumentation and mechanical alignment. A natural slope and rotation along the grinding marks on the arthritic tibial plateau is aimed for, followed by resection of the tibial plateau. After determining the femoral rotation with the intramedullary balancer, the distal femur is resected first (extension gap). This is followed by a posterior (flexion gap) and anterior femoral condylar resection.

All TKAs were performed between January 2017 and December 2020 by MPA (CIM and OTS) and by TR and RK (OTS). All surgeons had many years of experience in TKA and a high volume of operations. The same perioperative and postoperative anaesthesia and pain management protocol were used for all patients as well as a medial parapatellar approach without tourniquet. The postoperative rehabilitation protocol was the same for all patients and included immediate full weight-bearing on crutches until sufficient muscular stabilisation was achieved.

### Data collection

Data were collected during routine visits before the surgery, after 4 months, 1 year and 2 years using Research Electronic Data Capture (REDCap^®^). Table 1 provides a detailed overview of the measures and data collection. Patients’ characteristics were extracted from the medical records. Osteoarthritis was classified according to Kellgren and Lawrence (KL) grade from 0 (no osteoarthritis) to 4 (severe osteoarthritis) [30] and comorbidities according to the American Society of Anesthesiologists (ASA) from ASA I (normal healthy) to ASA V (moribund) [31].

**Table 1:** Measures and data collection

Measure and scale	Data collection			
	Before	4 months	1 year	2 years
*PROM*				
Patient satisfaction, five-point Likert scale very satisfied, satisfied, neutral, unsatisfied, very unsatisfied		x	x	x
Overall improvement, seven-point Likert scale very much better, substantially better, a little better, no change, a little worse, substantially worse, very much worse			x	x
Surgery again Yes, no			x	x
KOOS [51] pain, symptoms, daily living, sports, quality of life 0 (worst) to 100 (best) points	x	x	x	x
FJS-12 [52], ability to forget the artificial joint in everyday life 0 (worst) to 100 (best) points	x	x	x	x
HAAS [53], high-intensity activities 0 (worst) to 18 (best) points		x	x	x
EQ-5D-3L [54], health-related quality of life 0 (worst) to 1 (best)	x	x	x	x
EQ-VAS [54], health-related quality of life 0 (worst) to 100 (best)	x	x	x	x
*Surgeon reported*				
KSS-Knee [55], objective knee function 0 (worst) to 100 (best) points	x	x	x	
Surgeon satisfaction, five-point Likert scale very satisfied, satisfied, neutral, unsatisfied, very unsatisfied		x	x	

*PROM* patient-reported outcome measure, *KOOS* Knee injury and Osteoarthritis Outcome Score, *FJS-12* Forgotten Joint Score, *HAAS* High-Activity Arthroplasty Score, *VAS* Visual Analogue Scale, *KSS* Knee Society Score

The primary outcome was patient satisfaction on a five-point Likert scale. Patients were summarised as satisfied (‘very satisfied’ or ‘satisfied’) and not satisfied (‘neutral’, ‘unsatisfied’ or ‘very unsatisfied’). Secondary outcomes were all other PROMs: overall improvement (‘very much better’ or ‘substantially better’ were summarised as improved, the rest as not improved), the willingness to undergo the surgery again, the Knee injury and Osteoarthritis Outcome Score (KOOS), the Forgotten Joint Score (FJS-12), the High-Activity Arthroplasty Score (HAAS) and the EQ-5D-3L for health-related quality of life including a visual analogue scale (VAS).

In addition, surgeons completed the objective part of the Knee Society Score (KSS), also known as KSS-Knee, and rated their satisfaction with the surgery. Similar to patient satisfaction, ‘very satisfied’ and ‘satisfied’ were combined as satisfied. The KSS was not available after 2 years, because it required a follow-up visit, which was not routine for all patients.

Postoperative complications such as thromboembolic event, infection, re-operation, revision or decease were recorded as adverse events. Revision was defined as a re-operation to replace some or all parts of the original TKA.

### Sample size and matching

**Fig. 1:**
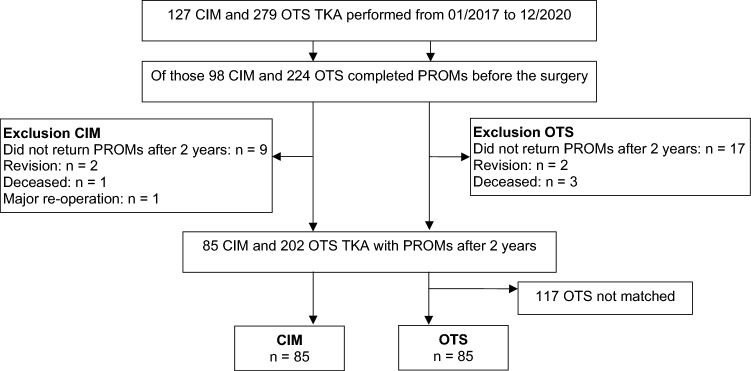
Flow chart of recruitment. *CIM* customised individually made, *OTS* off-the-shelf, *TKA* total knee arthroplasty, *PROMs* patient-reported outcome measures, *n* number of patients

The a priori power calculation was based on a calculated mean effect size of 0.5 across all measures. This resulted in a sample size of 85 TKAs per group to assure a power of 0.9 with a two-sided alpha of 0.05. To reduce the bias introduced by the non-randomised study design and to adjust for differences in patients’ characteristics, we performed a propensity score matching based on the variables age, body mass index (BMI), sex, KL grade and ASA score. Of 85 CIM and 202 OTS TKA with available 2-year PROMs, 85 CIM were matched to 85 OTS TKA (Fig. 1).

### Statistics

Descriptive statistics comprise mean and standard deviation (SD) for continuous variables, frequency count and percentage for categorical variables. Differences between preoperative and postoperative data were measured with paired *t* test. Differences between groups were measured with unpaired *t* test for continuous variables and with Mann–Whitney U test or Chi-square test for categorical variables. Bivariate linear correlations were analysed using the Spearman test, with effect sizes interpreted as low (*r* ≈ 0.1), medium (*r* ≈ 0.3) or strong (*r* ≈ 0.5) [32]. Statistical analyses were performed using IBM SPSS statistics for Windows, version 29, Armonk, NY: IBM Corp and R, version 4.1.3 [33]. Matching was performed using the MatchIt package in R, version 4.5.3.

## Results

### Recruitment and baseline measures

The matched-pair data of 85 CIM TKA (70 patients, 34 women) and 85 OTS TKA (78 patients, 33 women) was analysed. Details to recruitment are described in Fig. 1 and patients’ characteristics in Table 2. Patients with CIM TKA had more often a supplementary insurance which is required in Switzerland to cover costs for a CIM TKA. Patients with CIM TKA had more often a staged bilateral surgery and at baseline higher PROMs and a lower KSS (Table 2).

**Table 2:** Patients’ characteristics and baseline measures

	CIM *n* = 85	OTS *n* = 85	Difference	
	Mean (± SD)	Mean (± SD)	*p* value	[95% CI]
*Patients’ characteristics*				
Age, years	66.7 (± 8.6)	66.3 (± 9.1)	0.792	[− 2.3 to 3.0]
BMI, kg/m^2^	26.4 (± 3.2)	26.7 (± 3.9)	0.617	[− 1.4 to 0.8]
Sex, *n* (%)			0.756	
Women	37 (44%)	34 (40%)		
Men	48 (56%)	51 (60%)		
Insurance, *n* (%)			< 0.001	
Basic	5 (6%)	57 (67%)		
Supplementary	80 (94%)	28 (33%)		
Side, *n* (%)			0.575	
Left	36 (42%)	39 (46%)		
Right	49 (58%)	46 (54%)		
Surgery, *n* (%)			0.008	
Unilateral	55 (65%)	71 (84%)		
Bilateral	30 (35%)	14 (16%)		
KL grade, *n* (%)			0.857	
2			1 (1%)	
3	19 (22%)	20 (24%)		
4	66 (78%)	64 (75%)		
ASA classification, *n* (%)			0.494	
I/II	76 (89%)	72 (85%)		
III	9 (11%)	13 (15%)		
Length of stay, days	6.1 (± 1.2)	6.3 (± 1.1)	0.375	[− 0.5 to 0.2]
*Baseline measures*				
KOOS symptoms	51.5 (± 17.1)	47.0 (± 20.2)	0.123	[− 1.2 to 10.1]
KOOS pain	50.2 (± 16.2)	43.2 (± 15.4)	0.004	[2.2 to 11.8]
KOOS daily living	58.2 (± 15.6)	51.2 (± 18.4)	0.009	[1.8 to 12.1]
KOOS sports	24.0 (± 16.1)	20.0 (± 16.6)	0.124	[− 1.1 to 9.1]
KOOS quality of life	26.8 (± 12.9)	25.1 (± 14.3)	0.421	[− 2.4 to 5.8]
FJS-12	18.0 (± 12.3)	15.4 (± 13.3)	0.203	[− 1.4 to 6.5]
HAAS (not administered)	–	–		
EQ-5D-3L	0.65 (± 0.16)	0.62 (± 0.18)	0.269	[− 0.02 to 0.08]
EQ-VAS	65.5 (± 21.8)	60.0 (± 22.2)	0.115	[− 1.3 to 12.2]
KSS	53.1 (± 11.4)	58.0 (± 13.3)	0.010	[− 8.7 to − 1.2]

*CIM* customised individually made, *OTS* off-the-shelf, *n* number of patients, *SD* standard deviation, *BMI* body mass index, *KL* Kellgren and Lawrence grade of osteoarthritis, *ASA* American Society of Anesthesiologists, *KOOS* Knee injury and Osteoarthritis Outcome Score, *FJS-12* Forgotten Joint Score, *HAAS* High-Activity Arthroplasty Score, *VAS* Visual Analogue Scale, *KSS* Knee Society Score

### Postoperative measures

#### PROMs

**Fig. 2:**
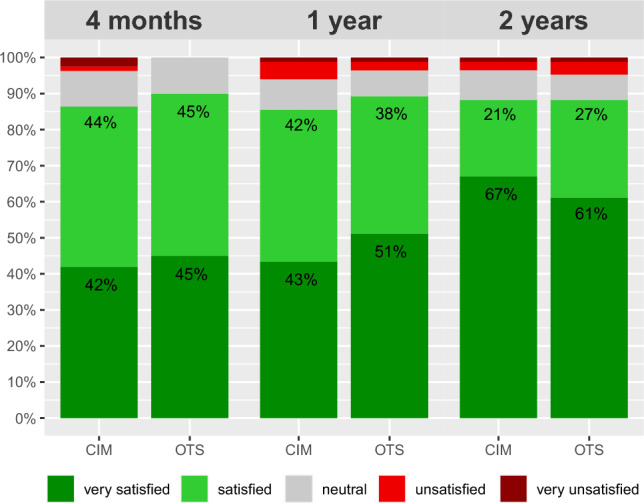
Patient satisfaction at follow-up. *CIM* customised individually made, *OTS* off-the-shelf

Patient satisfaction after 2 years was 88% for CIM and OTS TKA (Table 3 and Fig. 2). Overall, eight patients (5%) were not satisfied after 1 year but were satisfied after 2 years and seven patients (4%) were satisfied after 1 year but not satisfied after 2 years. All other patients (91%) had no change in patient satisfaction. Almost all patients reported an overall improvement and would undergo the surgery again (Table 3). All other PROMs improved for all patients from baseline to each follow-up (4 months, 1 year and 2 years), as well as from 4 months to 1 year and from 1 to 2 years (*p* < 0.001 each). Sole exception was the EQ-VAS with a mean change of − 0.7 from 1 to 2 years (*p* = 0.218).

**Table 3:** Postoperative outcome measures of patients with CIM and OTS TKA

	CIM *n* = 85	OTS *n* = 85	Difference	
	Mean (± SD)	Mean (± SD)	*p* value	[95% CI]
*4 months*				
Satisfied patient, *n* (%)	70 (86%)	72 (90%)	0.725	
KOOS symptoms	67.3 (± 16.1)	68.4 (± 16.4)	0.676	[− 3.9 to 6.0]
KOOS pain	70.8 (± 16.1)	70.4 (± 16.9)	0.894	[− 5.4 to 4.7]
KOOS daily living	78.7 (± 14.1)	78.7 (± 14.5)	0.997	[− 4.3 to 4.3]
KOOS sports	48.9 (± 23.7)	53.8 (± 23.0)	0.208	[− 2.8 to 12.7]
KOOS quality of life	56.2 (± 20.4)	57.1 (± 20.3)	0.763	[− 5.3 to 7.2]
FJS-12	47.6 (± 25.7)	44.8 (± 25.8)	0.481	[− 10.8 to 5.1]
HAAS	10.4 (± 2.8)	9.8 (± 2.3)	0.288	[− 1.6 to 0.5]
EQ-5D-3L	0.83 (± 0.15)	0.79 (± 0.15	0.105	[− 0.08 to 0.01]
EQ-VAS	79.7 (± 13.1)	72.1 (± 18.19	0.003	[− 12.5 to − 2.7]
KSS	90.9 (± 6.6)	85.0 (± 8.9)	< 0.001	[− 8.3 to − 3.5]
Satisfied surgeon, *n* (%)	75 (91%)	76 (92%)	0.753	
*1 year*				
Satisfied patient, *n* (%)	71 (86%)	75 (89%)	0.844	
Improved patient, *n* (%)	63 (83%)	64 (88%)	0.643	
Surgery again, *n* (%)	70 (92%)	69 (96%)	0.496	
KOOS symptoms	75.3 (± 17.0)	80.4 (± 15.5)	0.043	[0.2 to 10.1]
KOOS pain	81.9 (± 16.6)	83.9 (± 15.2)	0.420	[− 2.9 to 6.8]
KOOS daily living	86.3 (± 13.7)	86.1 (± 14.4)	0.939	[− 4.5 to 4.1]
KOOS sports	66.0 (± 21.5)	64.9 (± 24.7)	0.758	[− 8.5 to 6.2]
KOOS quality of life	69.8 (± 21.4)	71.3 (± 21.8)	0.654	[− 5.1 to 8.1]
FJS-12	65.0 (± 25.5)	65.4 (± 26.4)	0.913	[− 7.5 to 8.4]
HAAS	12.3 (± 2.6)	11.2 (± 2.4)	0.016	[− 2.0 to − 0.2]
EQ-5D-3L	0.87 (± 0.14)	0.87 (± 0.13)	0.562	[− 0.03 to 0.05]
EQ-VAS	81.4 (± 14.7)	80.2 (± 13.5)	0.606	[− 5.5 to 3.1]
KSS	94.6 (± 6.1)	89.0 (± 8.0)	< 0.001	[− 8.0 to − 3.4]
Satisfied surgeon, *n* (%)	75 (96%)	70 (92%)	0.382	
*2 years*				
Satisfied patient, *n* (%)	75 (88%)	75 (88%)	0.883	
Improved patient, *n* (%)	78 (92%)	76 (89%)	0.890	
Surgery again, *n* (%)	75 (90%)	77 (96%)	0.211	
KOOS symptoms	80.8 (± 14.8)	83.4 (± 16.6)	0.293	[− 2.2 to 7.3]
KOOS pain	87.1 (± 14.7)	86.2 (± 17.5)	0.720	[− 5.8 to 4.0]
KOOS daily living	90.6 (± 12.3)	89.1 (± 14.5)	0.463	[− 5.6 to 2.6]
KOOS sports	69.9 (± 21.6)	72.0 (± 22.5)	0.563	[− 5.0 to 9.1]
KOOS quality of life	76.2 (± 21.2)	76.3 (± 22.5)	0.991	[− 6.6 to 6.7]
FJS-12	72.7 (± 23.5)	70.8 (± 26.6)	0.621	[− 9.5 to 5.7]
HAAS	12.9 (± 2.6)	11.7 (± 2.6)	0.004	[− 2.0 to − 0.4]
EQ-5D-3L	0.93 (± 0.12)	0.91 (± 0.13)	0.254	[− 0.06 to 0.02]
EQ-VAS	81.5 (± 15.7)	79.9 (± 14.8)	0.487	[− 6.3 to 3.0]

*CIM* customised individually made, *OTS* off-the-shelf, *n* number of patients, *SD* standard deviation, *CI* confidence interval, *KOOS* Knee injury and Osteoarthritis Outcome Score, *FJS-12* Forgotten Joint Score, *HAAS* High-Activity Arthroplasty Score, *VAS* Visual Analogue Scale, *KSS* Knee Society Score

**Fig. 3:**
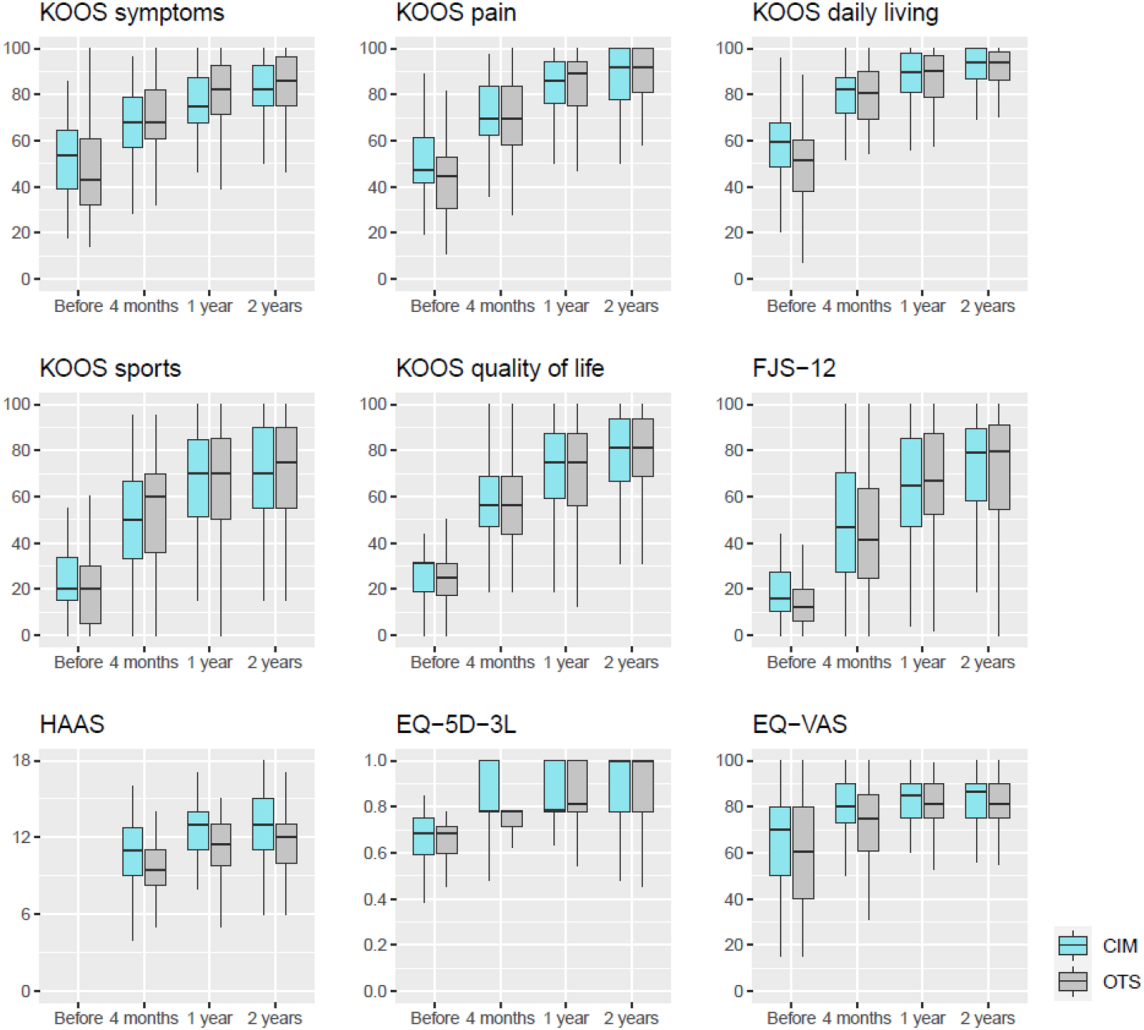
Boxplots of PROMs for CIM and OTS TKA. *CIM* customised individually made, *OTS* off-the-shelf, *KOOS* Knee injury and Osteoarthritis Outcome Score, *FJS-12* Forgotten Joint Score, *HAAS* High-Activity Arthroplasty Score, *VAS* Visual Analogue Scale

When comparing patients with CIM and OTS TKA, the EQ-VAS after 4 months and the HAAS after 1 year and 2 years were clearly higher for patients with CIM TKA (Table 3, Fig. 3). All other PROMs were not different in their end scores. Change scores of PROMs were higher for patients with OTS TKA from baseline to each follow-up with clearly higher values for KOOS symptoms, pain and daily living (Table 5, additional material).

#### KSS and surgeon satisfaction

The KSS improved for all patients from baseline to 4 months and from baseline to 1 year (*p* < 0.001). KSS end and change scores were higher for patients with CIM TKA (*p* < 0.001, Table 3 and additional material: Table 5). Surgeon satisfaction after 1 year was 96% for CIM and 92% for OTS TKA (*p* = 0.382, Table 3). The correlation between patient and surgeon satisfaction was strong (4 months: *r* = 0.418, *p* < 0.001; 1 year: *r* = 0.483, *p* < 0.001).

#### Satisfied compared to not satisfied patients

**Fig. 4:**
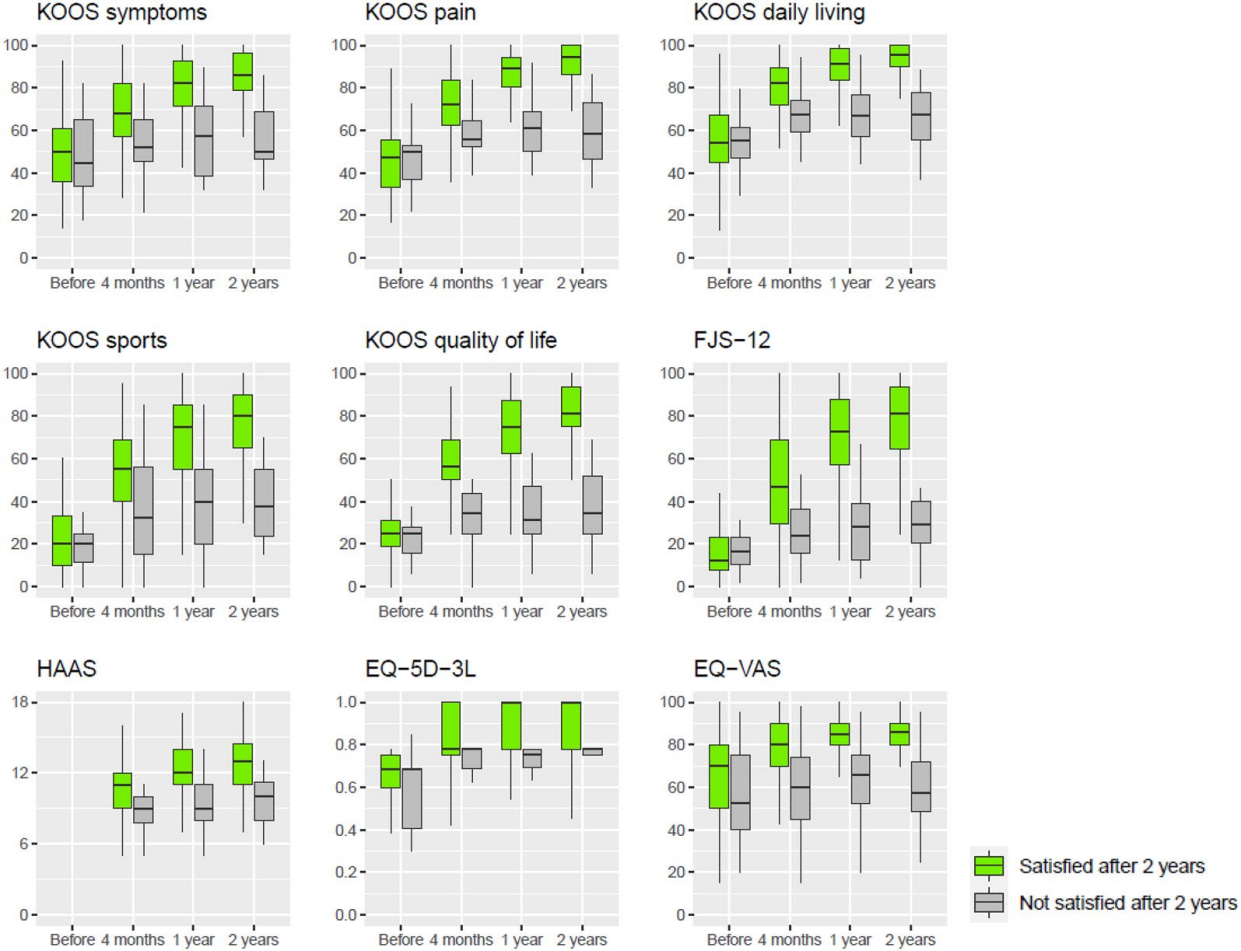
Boxplots of PROMs for satisfied and not satisfied patients after 2 years. *KOOS* Knee injury and Osteoarthritis Outcome Score, *FJS-12* Forgotten Joint Score, *HAAS* High-Activity Arthroplasty Score, *VAS* Visual Analogue Scale

Patients who were satisfied after 2 years did not differ at baseline from patients who were not satisfied (Table 4). At each follow-up, all PROMs and the KSS were higher for patients who were satisfied after 2 years (Table 4; Fig. 4). Likewise, the change scores for all PROMs and the KSS were higher for satisfied patients (additional material: Table 6).

**Table 4:** Comparison of satisfied and not satisfied patients at 2-year follow-up

	Baseline		*p*	4 months		*p*	1 year		*p*	2 years		*p*
	Satisfied at 2 years *n* = 150	Not satisfied at 2 years *n* = 20		Satisfied at 2 years *n* = 150	Not satisfied at 2 years *n* = 20		Satisfied at 2 years *n* = 150	Not satisfied at 2 years *n* = 20		Satisfied at 2 years *n* = 150	Not satisfied at 2 years *n* = 20	
	Mean (± SD)	Mean (± SD)		Mean (± SD)	Mean (± SD)		Mean (± SD)	Mean (± SD)		Mean (± SD)	Mean (± SD)	
*Patients’ characteristics*												
Age, years	66.3 (± 8.7)	67.6 (± 10.3)	0.564									
BMI, kg/m^2^	26.5 (± 3.6)	26.8 (± 3.5)	0.771									
Women, *n* (%)	61 (41%)	10 (50%)	0.474									
Basic insurance, *n* (%)	56 (37%)	6 (30%)	0.625									
Unilateral TKA, *n* (%)	110 (73%)	16 (80%)	0.600									
CIM TKA, *n* (%)	75 (50%)	10 (55%)	1.000									
KL grade 4, *n* (%)	116 (77%)	14 (70%)	0.480									
ASA I/II, *n* (%)	132 (88%)	16 (80%)	0.318									
Length of stay, days	6.2 (± 1.1)	6.6 (± 1.3)	0.148									
*Measures*												
Satisfied patient, *n* (%)	–	–		131 (92%)	11 (58%)	0.001	139 (95%)	7 (35%)	< 0.001			
Improved patient, *n* (%)	–	–		–	–		120 (93%)	7 (35%)	< 0.001	148 (99%)	6 (30%)	< 0.001
Surgery again, *n* (%)	–	–		–	–		126 (98%)	13 (68%)	< 0.001	144 (99%)	8 (44%)	< 0.001
KOOS symptoms	49.4 (± 18.6)	48.2 (± 20.8)	0.792	69.8 (± 15.2)	53.2 (± 16.2)	< 0.001	80.7 (± 14.0)	57.0 (± 18.1)	< 0.001	85.6 (± 11.8)	55.7 (± 16.4)	< 0.001
KOOS pain	46.7 (± 16.0)	47.0 (± 17.3)	0.945	72.2 (± 15.8)	58.9 (± 16.6)	< 0.001	85.9 (± 13.6)	60.8 (± 14.2)	< 0.001	90.3 (± 12.3)	59.7 (± 15.5)	< 0.001
KOOS daily living	54.8 (± 17.1)	53.9 (± 19.5)	0.836	80.4 (± 12.9)	65.9 (± 17.2)	< 0.001	88.8 (± 11.6)	67.0 (± 15.5)	< 0.001	93.1 (± 9.1)	65.7 (± 15.8)	< 0.001
KOOS sports	22.0 (± 16.2)	21.9 (± 18.7)	0.984	53.8 (± 22.0)	33.7 (± 25.9)	< 0.001	69.4 (± 20.3)	38.7 (± 22.8)	< 0.001	75.7 (± 18.2)	38.6 (± 18.3)	< 0.001
KOOS quality of life	26.1 (± 13.5)	24.7 (± 14.7)	0.682	59.2 (± 19.0)	38.1 (± 20.6)	< 0.001	75.1 (± 17.7)	37.5 (± 18.2)	< 0.001	81.6 (± 16.2)	36.6 (± 16.5)	< 0.001
FJS-12	16.5 (± 12.5)	18.5 (± 15.4)	0.591	48.8 (± 25.4)	28.0 (± 20.5)	< 0.001	70.1 (± 22.6)	29.1 (± 19.1)	< 0.001	77.4 (± 20)	29.9 (± 18)	< 0.001
HAAS^a^	–	–		10.3 (± 2.6)	8.6 (1.9)	0.028	12.2 (± 2.3)	9.2 (± 2.5)	< 0.001	12.7 (± 2.5)	9.9 (± 2.3)	< 0.001
EQ-5D-3L	0.64 (± 0.17)	0.59 (± 0.16)	0.242	0.82 (± 0.15)	0.75 (± 0.16)	0.077	0.89 (± 0.12)	0.75 (± 0.16)	< 0.001	0.94 (± 0.11)	0.78 (± 0.13)	< 0.001
EQ-VAS	63.6 (± 22.0)	56.3 (± 22.3)	0.206	78.4 (± 13.6)	57.1 (± 21.4)	< 0.001	83.0 (± 11.8)	64.1 (± 18.0)	< 0.001	83.8 (± 11.8)	58.1 (± 18.7)	< 0.001
KSS^a^	55.2 (± 12.7)	58.2 (± 11.3)	0.332	88.4 (± 8.5)	84.7 (± 6.5)	0.061	92.8 (± 7.2)	84.4 (± 6.5)	< 0.001	–	–	
Satisfied surgeon, *n* (%)	–	–		135 (93%)	16 (80%)	0.120	131 (98%)	14 (70%)	< 0.001	–	–	

*n* number of patients, *SD* standard deviation, *BMI* body mass index, *TKA* total knee arthroplasty, *CIM* customised individually made, *KL* Kellgren and Lawrence grade of osteoarthritis, *ASA* American Society of Anesthesiologists score for comorbidity, *KOOS* Knee injury and Osteoarthritis Outcome Score, *FJS-12* Forgotten Joint Score, *HAAS* High-Activity Arthroplasty Score, *VAS* Visual Analogue Scale, *KSS* Knee Society Score

^a^HAAS not administered at baseline, KSS not administered at 2-year follow-up

Patient satisfaction was not correlated with patients’ characteristics (age, BMI, sex, insurance, side, bilateral surgery, KL grade, ASA), implant or baseline measures. The correlation between patient satisfaction and measures after 1 year was medium for HAAS (*r* = 0.365, *p* < 0.001) and strong for KOOS, FJS-12, EQ-5D-3L, EQ-VAS and KSS (*r* > 0.411, *p* < 0.001). The correlation between patient satisfaction and measures after 2 years was medium for HAAS (*r* = 0.356, *p* < 0.001) and EQ-VAS (*r* = 0.333, *p* < 0.001) and strong for KOOS, FJS-12, EQ-5D-3L (*r* > 0.432, *p* < 0.001).

### Adverse events

At the last follow-up, 1 patient with CIM TKA and 3 patients with OTS TKA had died. Four revisions occurred: 2 CIM TKA after 17 and 26 months and 2 OTS TKS after 8 and 9 months, respectively. The revision rate was 2.4% in both groups. One patient with CIM TKA needed a major re-operation due to a quadriceps rupture after 19 months. These patients were excluded from the analysis (Fig. 1).

Of the patients included in the matched-pair analysis, 3 patients with CIM TKA and 1 patient with OTS TKA had an adverse event. Two patients, 1 with CIM and 1 with OTS TKA, required diagnostic arthroscopy to exclude an infection (both negative) and 2 patients with CIM TKA required arthrolysis.

## Discussion

The most important finding was that patient satisfaction after 2 years was high and not different between patients with CIM and OTS TKA. Thus, our hypothesis was not confirmed. Preoperatively, patients with a CIM TKA tended to have less subjective impairment and presented with higher PROMs. Postoperatively, patients with CIM TKA had a higher EQ-VAS after 4 months and a higher HAAS after 1 year and 2 years. All other PROMs were not different regarding the end scores between CIM and OTS TKA. The change scores of PROMs were higher for OTS TKA, especially for KOOS symptoms, pain and daily living.

The objective KSS was higher postoperatively for CIM TKA. Surgeon satisfaction was not different between CIM and OTS TKA and was strongly correlated with patient satisfaction. Patients who were satisfied after 2 years were clearly better on all PROMs and the KSS compared to patients who were not satisfied after 2 years.

Our results regarding patient satisfaction are within the spectrum of current TKA studies or registry reports [4, 10, 34, 35]. The results are also consistent with other CIM TKA studies. The largest retrospective study to date included 540 CIM TKA and found a satisfaction rate of 89% after a mean follow-up of 2.8 years (range 0.1–7.0) [22]. The authors reported a KOOS for Joint Replacement (KOOS-JR) of 82 points and a revision rate of 1.5%. The only study to date with a long-term follow-up found very good and stable results over 5 years [20]. Patient satisfaction was not analysed, but they found a mean KSS of 92 points, a mean WOMAC of 11 points and a revision rate 1.4% after 5 years. A study with posterior-stabilised CIM TKA (iTotal^®^ PS, Conformis Inc., Billerica, MA, US) reported a high satisfaction rate of 90% for 100 CIM TKA after a mean follow-up of 1.9 years (range 1.5–2.4) [36].

Comparative CIM TKA studies are still sparse. Our own group found no differences in patient satisfaction and other PROMs after 1 year in an unmatched comparison of 74 CIM and 169 OTS TKA [37]. Satisfaction rates were similar to the present study (CIM 87%, OTS 89%). Others found better clinical outcome and higher fulfilment of expectations for patients with CIM TKA after 1 year, although in a small sample of 33 CIM and 31 OTS TKA [38]. Another study examined PROMs of 47 CIM and 47 OTS TKA in the same patients with staged bilateral surgery. After a mean follow-up of 2.3 years (range 0.7–3.8), they found better results for CIM TKA regarding KOOS-JR, FJS-12, pain, mobility, stability and normal feeling of the knee. In summary, 72% of the patients preferred the CIM TKA, 21% saw no difference and 6% preferred the OTS TKA [39].

The strong correlation between patient satisfaction and PROMs at follow-up is consistent with other studies [4, 40]. In contrast to others, there was no correlation between dissatisfaction and younger age [4, 5, 9], higher BMI [4, 8], female sex [8] or low preoperative PROMs [4].

Most of the improvement in all PROMs and the KSS occurred quite early, within the first 4 months. By the 4-month follow-up, we found a clear difference in all measures for patients who were later satisfied and those who were not. Others also reported early different satisfaction profiles as early as 6 weeks [40] or after 3 months [41]. PROMs could support the early identification of dissatisfied patients and enable clinicians to intervene in a timely and targeted way to improve patient outcomes [40]. Nevertheless, all measures in our study improved considerably by the 2-year follow-up. However, the proportion of patients who went from being satisfied after 1 year to being not satisfied after 2 years, and vice versa, was rather small (9%). Others have also found no change in patient satisfaction from 6 months to 2 years [40] or only rare changes from 1 to 3 years [42].

As of 2018, another CIM TKA system is available, the Symbios Origin^®^ implant (Symbios, Yverdon-les-Bains, Switzerland) [43]. After promising first results [44], a large improvement in the KSS was recently shown, with a mean KSS of 94 points after 1 year [45]. Another study reported a high satisfaction rate of 94% after a mean follow-up of 2.8 years [46]. KOOS and FJS-12 results in this study were similar or slightly lower than our results after 2 years. Others found satisfactory early clinical and radiographic outcomes for this CIM TKA in patients with prior osteotomies or extra-articular fracture sequelae [47].

The strength of our study is the prospective matched-pair design which has not been previously published for CIM TKA. We applied a profound set of PROMs and analysed the data at multiple follow-ups, whilst having a reasonable number of drop-outs. Nevertheless, our study has some limitations. First, although the data were collected prospectively, a selection bias is possible due to the lack of randomisation. On the other hand, it must be recognised that patients in a private clinic setting would not accept this scientifically interesting randomisation. For practical reasons, this bias is, therefore, unavoidable. Selection bias also occurred because supplementary insurance is required to be eligible for CIM TKA.

Propensity score matching was used to limit bias and ensure a degree of homogeneity. The 2-year follow-up is only mid-term, but CIM TKAs are still relatively new and not widely used. However, for studies with PROMs as primary outcome, it was shown that a 1-year follow-up is sufficient, as results remain consistent with longer follow-up [48, 49]. Longer follow-up is preferable for implant survival. Our 2-year revision rate was 2.4% in both groups, which is lower than the reported overall 2-year revision rate of 3.5% reported in the Swiss Implant Registry (iTotal: 2.3%, Attune: 4.2%) [29]. The loss to follow-up of patients who did not return their PROMs questionnaire was 9% after 2 years. Despite constant efforts, including postal or e-mail reminders and telephone calls, achieving a high PROMs response rate at multiple time points has proven to be challenging [50].

## Conclusion

We found a high patient satisfaction after 1 year and after 2 years, which did not differ between patients with CIM and OTS TKA. The HAAS, which is designed to capture improvements in activities to recreational sports level, was superior for patients with CIM TKA. All other PROMs did not differ in terms of end scores. Change scores were higher for OTS TKA, especially for KOOS symptoms, pain and daily living. Both implant systems apparently improved function, pain and health-related quality of life.

### Supplementary Information

Below is the link to the electronic supplementary material.

Supplementary file1 (DOCX 34 kb)

Supplementary file2 (DOCX 35 kb)
