# Supplementary material for: Satisfaction after total knee arthroplasty: a prospective matched-pair analysis of patients with customised individually made and off-the-shelf implants
Source: Knee Surg Sports Traumatol Arthrosc. 2023 Nov 20;31(12):5873–84. doi: 10.1007/s00167-023-07643-1 (PMC10719143; doi:10.1007/s00167-023-07643-1)
Supplement: Supplementary file 1 — Supplementary file1 (DOCX 34 kb) [file 167_2023_7643_MOESM1_ESM.docx]

# Additional material

## Table 5: Changes of outcome measures for CIM and OTS TKA.

|  | CIM  n = 85 | |  | OTS  n = 85 | |  | Difference | |  |
| --- | --- | --- | --- | --- | --- | --- | --- | --- | --- |
|  | mean | (±SD) |  | mean | (±SD) |  | P value | [95% CI] |  |
| *Changes from baseline to 4 months* | | | | | | | | | |
| KOOS symptoms | 15.9 | (±22.7) |  | 21.1 | (±23.9) |  | .149 | [-12.4 to 1.9] | |
| KOOS pain | 20.3 | (±20.9) |  | 26.9 | (±19.4) |  | .037 | [-12.8 to -0.4] | |
| KOOS daily living | 20.0 | (±20.4) |  | 27.1 | (±19.2) |  | .023 | [-13.2 to -1.0] | |
| KOOS sports | 24.1 | (±28.8) |  | 33.8 | (±23.7) |  | .032 | [-18.7 to -0.8] | |
| KOOS quality of life | 29.3 | (±25.1) |  | 32.2 | (±22.2) |  | .442 | [-10.1 to 4.4] | |
| FJS-12 | 29.6 | (±29.4) |  | 29.2 | (±24.7) |  | .941 | [-8.2 to 8.8] | |
| EQ-5D-3L | 0.17 | (±0.19) |  | 0.18 | (±0.20) |  | .838 | [-0.07 to 0.05] | |
| EQ-VAS | 14.0 | (±24.0) |  | 12.1 | (±24.5) |  | .625 | [-5.8 to 9.5] | |
| KSS | 38.0 | (±12.8) |  | 26.6 | (±14.0) |  | < .001 | [7.2 to 15.5] | |
| *Changes from baseline to 1 year* | | | | | | | | | |
| KOOS symptoms | 24.3 | (±23.3) |  | 33.2 | (±21.9) |  | .011 | [-15.8 to -2.0] | |
| KOOS pain | 31.8 | (±21.4) |  | 40.5 | (±17.6) |  | .004 | [-14.8 to -2.8] | |
| KOOS daily living | 28.2 | (±18.7) |  | 34.6 | (±19.3) |  | .031 | [-12.3 to -0.6] | |
| KOOS sports | 42.1 | (±27.2) |  | 45.0 | (±24.7) |  | .497 | [-11.4 to 5.6] | |
| KOOS quality of life | 42.8 | (±24.5) |  | 46.8 | (±22.6) |  | .283 | [-11.2 to 3.3] | |
| FJS-12 | 46.5 | (±28.9) |  | 49.8 | (±26.5) |  | .441 | [-11.9 to 5.2] | |
| EQ-5D-3L | 0.21 | (±0.19) |  | 0.26 | (±0.17) |  | .137 | [-0.10 to 0.01] | |
| EQ-VAS | 16.0 | (±24.1) |  | 19.7 | (±21.7) |  | .300 | [-11.0 to 3.4] | |
| KSS | 41.1 | (±13.1) |  | 31.5 | (±14.8) |  | < .001 | [5.1 to 14.0] | |
| *Changes from baseline to 2 years* | | | | | | | | | |
| KOOS symptoms | 29.3 | (±23.2) |  | 36.3 | (±20.8) |  | .042 | [-13.7 to -0.3] | |
| KOOS pain | 36.9 | (±21.4) |  | 42.9 | (±18.5) |  | .053 | [-12.1 to 0.1] | |
| KOOS daily living | 32.5 | (±19.1) |  | 37.5 | (±18.9) |  | .087 | [-10.8 to 0.7] | |
| KOOS sports | 45.8 | (±26.3) |  | 50.5 | (±23.7) |  | .254 | [-13.0 to 3.5] | |
| KOOS quality of life | 49.4 | (±24.1) |  | 52.1 | (±22.3) |  | .461 | [-9.7 to 4.4] | |
| FJS-12 | 54.7 | (±28.4) |  | 55.8 | (±26.0) |  | .791 | [-9.5 to 7.3] | |
| EQ-5D-3L | 0.28 | (±0.19) |  | 0.29 | (±0.20) |  | .779 | [-0.07 to 0.05] | |
| EQ-VAS | 16.3 | (±24.8) |  | 19.9 | (±23.6) |  | .341 | [-11.1 to 3.9] | |

*CIM: customised individually made, OTS: off-the-shelf, n: number of patients, SD: standard deviation, CI: confidence interval, KOOS: Knee injury and Osteoarthritis Outcome Score, FJS-12: Forgotten Joint Score, VAS: Visual Analogue Scale, KSS: Knee Society Score*
