# Supplementary material for: Satisfaction after total knee arthroplasty: a prospective matched-pair analysis of patients with customised individually made and off-the-shelf implants
Source: Knee Surg Sports Traumatol Arthrosc. 2023 Nov 20;31(12):5873–84. doi: 10.1007/s00167-023-07643-1 (PMC10719143; doi:10.1007/s00167-023-07643-1)
Supplement: Supplementary file 2 — Supplementary file2 (DOCX 35 kb) [file 167_2023_7643_MOESM2_ESM.docx]

# Additional material

## Table 6: Changes of outcome measures for satisfied and not satisfied patients after two years.

|  | Satisfied  at 2 years  n = 150 | |  | Not satisfied  at 2 years  n = 20 | |  | Difference | |  |
| --- | --- | --- | --- | --- | --- | --- | --- | --- | --- |
|  | mean | (±SD) |  | mean | (±SD) |  | P value | [95% CI] |  |
| *Changes from baseline to 4 months* | | | | | | | | | |
| KOOS symptoms | 20.3 | (±22.4) |  | 5.0 | (±26.8) |  | .006 | [4.5 to 26.1] | |
| KOOS pain | 25.2 | (±19.6) |  | 11.9 | (±22.7) |  | .006 | [3.9 to 22.7] | |
| KOOS daily living | 25.1 | (±19.4) |  | 12.0 | (±21.5) |  | .016 | [2.6 to 23.6] | |
| KOOS sports | 31.1 | (±25.0) |  | 12.7 | (±33.6) |  | .007 | [5.0 to 31.8] | |
| KOOS quality of life | 33.0 | (±22.6) |  | 13.2 | (±24.7) |  | < .001 | [7.5 to 32.2] | |
| FJS-12 | 32.0 | (±25.9) |  | 10.1 | (±28.9) |  | < .001 | [9.2 to 34.5] | |
| EQ-5D-3L | 0.17 | (±0.20) |  | 0.18 | (±0.18) |  | .958 | [-0.10 to 0.09] | |
| EQ-VAS | 14.6 | (±22.5) |  | 1.1 | (±33.0) |  | .025 | [1.7 to 25.3] | |
| KSS | 33.0 | (±14.6) |  | 26.6 | (±12.5) |  | .061 | [-0.3 to 13.3] | |
| *Changes from baseline to 1 year* | | | | | | | | | |
| KOOS symptoms | 31.5 | (±21.3) |  | 8.8 | (±25.5) |  | < .001 | [12.5 to 33.0] | |
| KOOS pain | 39.2 | (±18.0) |  | 13.8 | (±20.9) |  | < .001 | [16.8 to 34.0] | |
| KOOS daily living | 33.9 | (±18.2) |  | 13.1 | (±16.7) |  | < .001 | [12.3 to 29.3] | |
| KOOS sports | 47.4 | (±23.3) |  | 18.0 | (±28.8) |  | < .001 | [17.6 to 41.0] | |
| KOOS quality of life | 48.8 | (±20.7) |  | 13.5 | (±20.9) |  | < .001 | [25.4 to 45.3] | |
| FJS-12 | 53.2 | (±24.3) |  | 10.1 | (±22.3) |  | < .001 | [31.4 to 54.7] | |
| EQ-5D-3L | 0.24 | (±0.18) |  | 0.16 | (±0.19) |  | .063 | [-0.00 to 0.17] | |
| EQ-VAS | 19.0 | (±21.6) |  | 8.3 | (±31.2) |  | .064 | [-0.6 to 21.9] | |
| KSS | 37.8 | (±14.4) |  | 26.2 | (±13.3) |  | < .001 | [4.9 to 18.4] | |
| *Changes from baseline to 2 years* | | | | | | | | | |
| KOOS symptoms | 36.2 | (±19.6) |  | 7.50 | (±24.9) |  | < .001 | [19.2 to 38.2] | |
| KOOS pain | 43.6 | (±16.8) |  | 12.8 | (±22.9) |  | < .001 | [22.5 to 39.1] | |
| KOOS daily living | 38.1 | (±16.7) |  | 11.8 | (±20.2) |  | < .001 | [18.2 to 34.3] | |
| KOOS sports | 52.8 | (±21.3) |  | 16.9 | (±26.1) |  | < .001 | [25.2 to 46.6] | |
| KOOS quality of life | 55.6 | (±18.4) |  | 12.5 | (±21.7) |  | < .001 | [34.1 to 52.1] | |
| FJS-12 | 60.9 | (±21.9) |  | 11.9 | (±25.0) |  | < .001 | [38.2 to 59.7] | |
| EQ-5D-3L | 0.29 | (±0.19) |  | 0.21 | (±0.18) |  | .068 | [-0.01 to 0.18] | |
| EQ-VAS | 19.9 | (±23.1) |  | 3.2 | (±28.1) |  | .005 | [5.1 to 28.5] | |

*n: number of patients, SD: standard deviation, CI: confidence interval, KOOS: Knee injury and Osteoarthritis Outcome Score, FJS-12: Forgotten Joint Score, VAS: Visual Analogue Scale, KSS: Knee Society Score*
